# Depression and the use of conversational AI for companionship among college students: the mediating role of loneliness and the moderating effects of gender and mind perception

**DOI:** 10.3389/fpubh.2025.1580826

**Published:** 2025-05-30

**Authors:** Lizu Lai, Yiyu Pan, Ranyuan Xu, Yanglang Jiang

**Affiliations:** ^1^School of Humanities, Hubei University of Chinese Medicine, Wuhan, China; ^2^Hubei Key Research Base of Humanities and Social Sciences, Hubei Health Industry Development Research Center, Wuhan, China

**Keywords:** depression, AI chatbots, companionship, loneliness, mind perception

## Abstract

**Introduction:**

The present study aimed to examine the relationship between depression and the use of conversational AI for companionship (UCAI-C) among college students. It further sought to investigate the mediating role of loneliness and the moderating roles of gender and mind perception in this association.

**Methods:**

A cross-sectional survey was conducted with 1,379 college students (Mean age = 21.93 years; 616 females, 763 males) using four validated instruments. Structural equation modeling was employed for data analysis.

**Results:**

Depression was found to be positively associated with UCAI-C. This relationship was significantly mediated by loneliness. Moreover, both gender and mind perception moderated the pathways between depression, loneliness, and UCAI-C.

**Conclusion:**

The findings illustrate how individuals’ mental states can influence their use of companion AI. They highlight significant individual differences (gender and mind perception) in these relationships, contributing to the theoretical understanding of factors governing human interaction with AI chatbots.

## 1 Introduction

Recent advancements in communicative artificial intelligence (AI) have paved the way for innovative approaches to mental health care, particularly through the deployment of social AI chatbots. Powered by sophisticated Large Language Models (LLMs) like GPT-4, these chatbots can mimic human behavior and become engaging and adaptive social companions (1). They are not only proficient in executing complex cognitive tasks but also excel at recognizing emotional states and generating empathetic responses (2). Substantial evidence suggests that AI-driven interactions can mirror the empathetic responses traditionally expected from human experts, providing a valuable resource in mental health support (3–5). Moreover, AI chatbots have proven effective in making users feel heard and understood, fulfilling a crucial component of companionship (6).

As these chatbots gain traction globally, their role extends beyond mere entertainment to address more profound psychological needs (7, 8). This shift is particularly relevant in the context of mental health (9). The market has seen a proliferation of chatbots specifically designed for mental health purposes and those capable of assuming various roles to offer companionship. Popular companion conversational AI such as Replika, Pi, Character.ai, XiaoIce, and DouBao are not only backed by substantial investment but are also witnessing an expanding user base, underscoring an increased acceptance of technology and a growing demand for digital companionship (10, 11). This trend highlights the significant impact of these technologies in enhancing daily social interactions and fulfilling emotional needs.

In light of these technological advancements, recent studies have explored the antecedents of AI acceptance and use across various technology acceptance models (12, 13). However, a significant gap remains in understanding the specific relationship between depression and the use of conversational AI for companionship (UCAI-C), particularly among college students who exhibit higher acceptance of new technology alongside notable mental health challenges (14).

Although there is currently limited empirical research specifically addressing the relationship between depression and UCAI-C, significant attention has been devoted to understanding the association between depression and other forms of technology, such as social media (15), screen media (16), and games (17, 18). While much of this research focuses on how problematic technology use may exacerbate depressive symptoms, evidence also indicates that depression can act as a predisposing factor for increased engagement with technology. For example, there were several longitudinal studies that have demonstrated that depressive mood positively predict increased social media use (19, 20).

According to the theory of compensatory internet use (21), individuals may engage in online activities, such as social media and AI chatbots, as a means to alleviate negative emotions or fulfill unmet psychosocial needs. Conversational AI, as cutting-edge technological tools, provide virtual social interactions and demonstrate a level of empathetic responsiveness (10). They can also be personalized by users to have unique personalities, voices, and even appearances, enhancing their utility as interactive companions (22). A person suffering from depressive symptoms might turn to conversational AI for comfort, understanding and advice. Given these capabilities, it is plausible to suggest that individuals with depression might seek out conversational AI as a supportive resource for psychological needs, motivated by the chatbots’ ability to emulate human-like companionship.

However, it is hypothesized that individuals with depressive symptoms are more likely to use conversational AI for companionship rather than for learning or other functional tasks. AI chatbots have a wide range of applications, among which emotional companionship and educational assistance are common among college students. Considering this, it is expected that individuals with depression would particularly value the emotional support provided by conversational AI, using them primarily for companionship. This preference aligns with the companion chatbots’ ability to offer sustained, interactive engagement and empathetic responses (23, 24), features that are crucial for those seeking to mitigate feelings of loneliness and isolation associated with depression (25).

Loneliness may serve as a mediating variable in the association between depression and UCAI-C. Loneliness was defined as an aversive affective state that occurs when people experience a discrepancy between the relationship they wish to have and how they are currently perceived (26). Not only is loneliness a unique risk factor to contribute to the development of depression (27), but individuals with depression are also likely to experience heightened feelings of loneliness (28). Longitudinal studies have demonstrated that depressive symptoms predict increased social and emotional loneliness over time (29, 30).

The sociocognitive model of loneliness, rooted in evolutionary theory, proposes that the uncomfortable feelings associated with state loneliness drive individuals to seek reconnection with others (31), a phenomenon often referred to as the reaffiliation motive (32). Individuals experiencing loneliness often feel dissatisfied with their existing social relationships, fostering a robust desire to establish new social connections and companionship. In contemporary settings, digital platforms frequently serve as venues for those feeling lonely to seek either active social interaction or passive engagement (33, 34). Notably, high levels of loneliness are linked with increased participation in virtual communities and forums (35) and engagement on video platforms conducive to parasocial relationships (36).

In this digital age, companion AI may be an innovative approach to mitigating loneliness (37). Interaction with AI chatbots is often less demanding and complex than engaging with real people, which can be particularly appealing to those grappling with loneliness. Furthermore, many AI chatbots are programmed to demonstrate empathy and offer advice, thereby satisfying users’ needs for belonging and social interaction (38). Empirical evidence supports this notion, showing that loneliness predicts the use of AI chatbots (14, 39).

However, the dynamics between depression, loneliness, and UCAI-C may be influenced by factors such as gender and individuals’ perceptions of the chatbots’ mental states. Research indicated significant gender differences in the acceptance and use of AI technologies, with men generally displaying higher acceptance and usage rates compared to women (40), which suggested that men might be more inclined to use conversational AI to satisfy psychological needs stemming from depression and loneliness.

The concept of mind perception refers to the attribution of human-like mental states to non-human agents (41). With advancements in Large Language Models, the anthropomorphism of conversational AI has increased, leading to a greater tendency for users to perceive these chatbots as having cognitive abilities and emotional states (42). During human-machine interactions, higher levels of mind perception might intensify individuals’ willingness to engage and potentially foster emotional connections with machines (43). Thus, it is posited that the level of mind perception also moderates the relationship between depression, loneliness, and UCAI-C.

Overall, the current exploration of the relationship between individual depression and UCAI-C, as well as the mechanisms underlying this process, remains limited. Focusing on the college student demographic, this study aims to investigate the relationship between depression and UCAI-C, how loneliness mediates this process, and how gender differences and perceptions of the chatbots’ minds influence these dynamics. This research seeks to elucidate the complex interplay between psychological factors and technology usage, potentially guiding the development of more effective digital interventions for mental health.

## 2 Methods

### 2.1 Participants and procedures

Participants were recruited via online advertisements in several universities in China. Informed consent was obtained from all participants, and the data collection process was conducted anonymously to safeguard participants’ privacy and mitigate potential social desirability bias. After applying predefined quality control criteria—such as excluding incomplete responses or those exhibiting patterned or inconsistent answers—a total of 1,379 participants’ data was included in analyses (616 females, 763 males). The mean age was 21.93 (range from 18 to 31, SD = 2.29).

### 2.2 Measures

#### 2.2.1 Patient health questionnaire (PHQ-9)

The PHQ-9 is a widely used tool for assessing the level of depression in individuals (44). It consists of 9 items that participants respond to their feelings and experiences over the past 2 weeks. Each item is scored on a scale from 0 to 3, where 0 means “not at all” and 3 means “nearly every day.” The total possible score on the PHQ-9 ranges from 0 to 27. Scores between 10 and 19 suggest moderate depression, while scores of 20 and above indicate the possibility of severe depression. This scale is valuable in both clinical and research settings for diagnosing depression and monitoring treatment response (45). In the present study, the Cronbach’s *α* was 0.903.

#### 2.2.2 University of California Los Angeles loneliness scale (UCLS-6)

The 6-item UCLAS is a simplified Chinese version of the UCLAS (46). Each item is rated on a 4-point Likert scale (1 = Never, 4 = Often). The total score ranges from 6 to 24, with higher scores indicating greater loneliness. The UCLS-6 has been demonstrated as an effective tool for measuring loneliness among Chinese adults. In the present study, the Cronbach’s *α* was 0.898.

#### 2.2.3 Mind perception scale

This concise scale includes 11 items to measure mind agency and mind experience towards AI chatbots (47, 48). Mind agency refers to the perceived capacity of the AI chatbots to recognize emotions, have thought, memory, self-control, plan and be moral. Mind experience is the perceived capacity of the AI chatbots to feel pleasure, hunger, pain, and have personality and consciousness. The possible range of scores was from 6 to 42 for mind agency, and from 5 to 35 for mind experience. The Cronbach’s *α* was 0.877 in this study.

#### 2.2.4 Conversational AI use behavior

The use behavior of conversational AI for learning and companionship was assessed separately using a 7-point scale item adapted from Strzelecki’s research. While Strzelecki’s original item specifically measured engagement frequency with ChatGPT, we modified it to assess the broader usage of conversational AI chatbots designed for learning and companionship purposes. Participants rated their frequency of interaction with AI chatbots from “1 = never” to “7 = several times a day,” providing a comprehensive measure of how often individuals engage with these tools in both educational and social-companion contexts. This modification allowed us to generalize beyond a single chatbot (e.g., ChatGPT) and capture a broader range of conversational AI interactions, such as those provided by companionship-specific platforms (e.g., Replika).

### 2.3 Statistical analyses

Descriptive statistics, correlation analysis, Harman’s single-factor test, and questionnaire reliability analysis were conducted using SPSS 21.0. Mediation effects and moderated mediation effects were tested using the bruceR package (49) in R 4.4.2. Initially, variables were centered, and under the control for age, the bias-corrected bootstrap method with 5,000 resamples was employed to estimate 95% confidence intervals.

## 3 Results

All data in this study were derived from the self-reports of the subjects, which may introduce common method bias. The Harman’s single-factor test was employed to assess this bias. The results showed that the first extracted common factor accounted for 27.61% of the variance, which is less than 40%. Therefore, there is no significant common method bias present.

The correlations between variables are shown in Table 1. Depression was significantly correlated with the UCAI-C (*r* = 0.170, *p* < 0.001), while there is no significant correlation with the use of AI chatbots for learning purposes.

**Table 1:** Descriptive statistics and Pearson correlation analysis results (*N* = 1,379).

Variable	*M* (SD)	Depression	Loneliness	UCAI-L	UCAI-C	Mind perception	Age	Gender
1. Depression	8.94 (6.17)	1						
2. Loneliness	13.76 (4.86)	0.658^***^	1					
3. UCAI-L	5.61 (1.56)	0.023	0.092^***^	1				
UCAI-C	4.16 (1.88)	0.170^***^	0.190^***^	0.405^***^	1			
Mind perception	28.59 (7.05)	0.243^***^	0.104^***^	0.264^***^	0.387^***^	1		
Age	21.93 (2.29)	0.185^***^	0.070^**^	−0.040	0.015	0.127^***^	1	
Gender	-	−0.089^***^	0.115^***^	0.119^***^	0.071^**^	0.061^*^	0.012	1

UCAI-L, the Use of conversational AI for learning; UCAI-C, the Use of conversational AI for companionship; ^*^p < 0.05, ^**^p < 0.01, ^***^p < 0.001.

**Figure 1:**
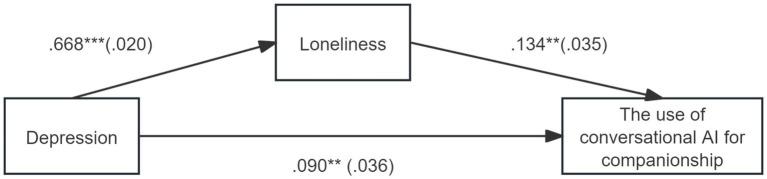
Mediation effects examined in this study.

The standardized coefficients of the hypothesized paths are depicted in Figure 1. Depression was positively associated with loneliness (*β* = 0.668, *SE* = 0.021, *p* < 0.001) and UCAI-C (*β* = 0.090, *SE* = 0.036, *p* < 0.05). Loneliness significantly predicted an increase of UCAI-C (*β* = 0.134, *SE* = 0.035, *p* < 0.001). The mediating effect of loneliness as a mediator between depression and UCAI-C was significant (*Estimate* = 0.089, *SE* = 0.025, *Z* = 3.607, *p* < 0.001). The mediating effect accounted for 49.72% of the total effect (*Estimate* = 0.179, *SE* = 0.026, *Z* = 6.911, *p* < 0.001). Even after excluding the mediating effects of loneliness, the direct effect from depression to UCAI-C was significant (*Estimate* = 0.090, *SE* = 0.036, *Z* = 2.460, *p* < 0.05).

To assess whether gender and mind perception moderates the indirect effect of depression on UCAI-C, a moderated mediation analysis was conducted (50). The results, presented in Table 2, demonstrated that both mind perception (*β* = 0.397, *SE* = 0.025, *p* < 0.001) and gender (*β* = 0.170, *SE* = 0.020, *p* < 0.01) significantly predicted UCAI-C. Additionally, interaction terms between gender and depression (*β* = 0.153, *SE* = 0.042, *p* < 0.001), as well as gender and loneliness (*β* = −0.112, *SE* = 0.044, *p* < 0.05), significantly predicted UCAI-C. Similarly, the interaction terms between mind perception and depression (*β* = 0.217, *SE* = 0.037, *p* < 0.001), and mind perception and loneliness (*β* = −0.155, *SE* = 0.036, *p* < 0.001), also significantly predicted UCAI-C. This pattern reveals the existence of a moderated mediation.

**Table 2:** Regression models.

Predictor outcome	(1) UCAI-C	(2) Loneliness	(3) UCAI-C
Age	−0.048 (0.027)	−0.035^*^ (0.020)	−0.068^**^ (0.025)
Depression	0.179^***^ (0.027)	0.691^***^ (0.021)	−0.101 (0.044)
Mind perception		−0.049^*^ (0.020)	0.397^***^ (0.025)
Gender		0.170^***^ (0.020)	0.081^**^ (0.025)
Loneliness			0.190^***^ (0.044)
Depression: Mind Perception			0.217^***^ (0.037)
Mind Perception: Loneliness			−0.155^***^ (0.036)
Depression: Gender			0.153^***^ (0.042)
Gender: Loneliness			−0.112^*^ (0.044)
*R* ^2^	0.031	0.467	0.210
Adj. *R*^2^	0.030	0.465	0.205
Num. obs.	1,379	1,379	1,379

Standardized regression coefficients are displayed, with standard errors in parentheses; UCAI-C, the Use of conversational AI for companionship; * *p* < 0.05. ** *p* < 0.01. *** *p* < 0.001.

**Figure 2:**
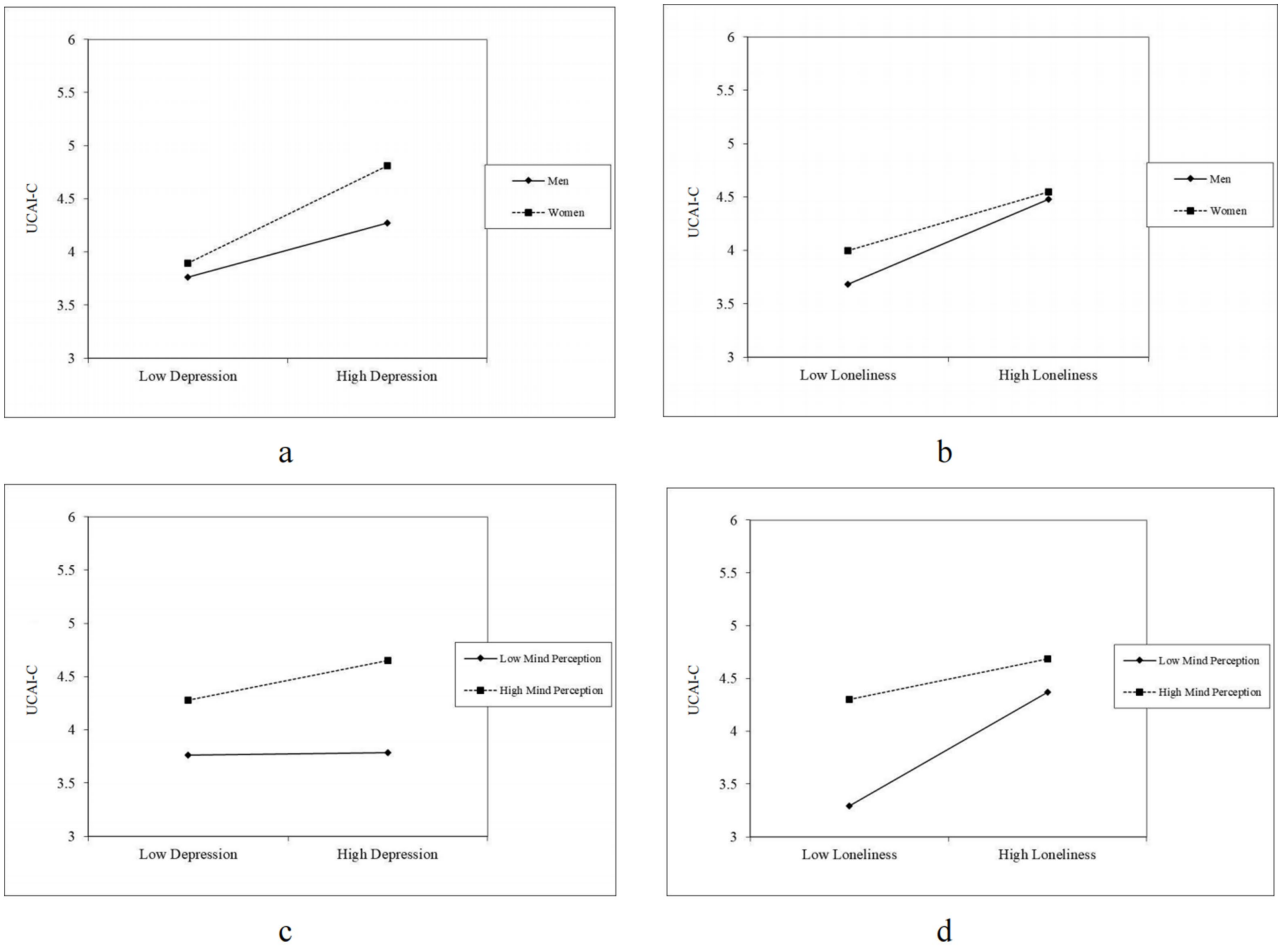
The moderating effects of gender and mind perception. **(a)** The moderating effect of gender on the association between depression and UCAI-C. **(b)** The moderating effect of gender on the association between loneliness and UCIA-C. **(c)** The moderating effects of mind perception on the relationship between depression and UCAI-C. **(d)** The moderating effect of mind perception on the relationship between loneliness and UCAI-C.

Further analysis of simple effects, as depicted in Figures 2a,b, revealed gender-specific differences. The relationship between depression and UCAI-C was stronger among females than among males. In contrast, the association between loneliness and UCAI-C was stronger among males than among males. Concerning conditional mediating effects, the mediating effect was significant among males (*Estimate* = 0.141, *SE* = 0.034, *Z* = 1.611, *95%CI*: 0.074, 0.206) but not females (*Estimate* = −0.014, *SE* = 0.040, *Z* = −0.356, *95%CI*: −0.097, 0.061).

Participants with high mind perceptions of conversational AI exhibited a positive direct effect of depression on UCAI-C; however, with lower mind perception of conversational AI, the predictive effect of loneliness on UCAI-C was stronger, as depicted in Figures 2c,d. Under conditions of low mind perception (−1 SD), the mediating effect was stronger (*Estimate* = 0.172, *SE* = 0.030, *Z* = 5.735, *p* < 0.001) (see Table 3).

**Table 3:** Conditional indirect effect.

Moderator	Level	*Estimate*	*SE*	*Z*	*95% CI*
Gender	Male	0.141	0.034	4.161^***^	0.074, 0.206
	Female	−0.014	0.040	−0.356	−0.097, 0.061
Mind perception	−1 SD MP	0.172	0.030	5.735^***^	0.114, 0.229
	Mean MP	0.094	0.022	4.177^***^	0.051, 0.137
	+1 SD MP	−0.000	0.035	−0.010	−0.067, 0.068

*** *p* < 0.001.

## 4 Discussion

The advancement of large language models has substantially enhanced the deployment of companion AI technologies. Exploring the dynamics between individual mental states, such as depression, and the utilization of companion AI provides valuable insights into the intricacies of human-machine interactions. The results of this investigation demonstrated a significant correlation between depression and UCAI-C, with loneliness acting as a mediating variable. Furthermore, gender and the perceived mind-like qualities of conversational AI significantly moderated these relationships. This study enriches our understanding of the nuanced factors that shape interactions between humans and AI, highlighting the importance of personal differences in these technological engagements.

In the current study, results revealed that depression significantly correlated with UCAI-C, but not with UCAI-L. This observation implies that individuals with depression predominantly seek emotional comfort and understanding from conversational AI, rather than pursuing knowledge acquisition or skill enhancement. Supporting literature suggests that daily stress positively predicts individuals’ pursuit of social support via platforms like Facebook (51), and social anxiety is linked to problematic use of conversational AI (39). Individuals experiencing negative emotions such as depression, anxiety, and stress tend to be uncomfortable with face-to-face interactions and encounter greater interpersonal challenges (52, 53), often resorting to online communication as a compensatory mechanism (54).

Loneliness mediated the relationship between depression and UCAI-C, indicating that higher levels of depression were associated with increased feelings of loneliness, which in turn led individuals to more frequently engage with conversational AI for companionship. Depression often led individuals to experience increased feelings of loneliness due to symptoms such as pessimism, low self-esteem, and social withdrawal, which in turn weakened social connections and satisfaction (55). In this context, loneliness may have prompted individuals to seek solace through AI chatbots as a means to alleviate these discomforts (56). Given that conversational AI provide immediate feedback and social interaction—even if superficial—this interaction could offer some relief from the loneliness exacerbated by depression (57). Recent research by Maples et al. (58) further supports the notion that university students experiencing loneliness actively turn to GPT3-enabled chatbots for emotional support and social connection, demonstrating the practical relevance of AI chatbots in mitigating loneliness and associated mental health risks among college populations (59).

When analyzing the moderating role of gender, distinct differences emerged between males and females in the relationship between loneliness, depression, and UCAI-C. According to the findings, male users were more inclined to utilize UCAI-C when experiencing loneliness, which may relate to the societal expectation for males to express emotional needs less frequently (60, 61). Traditionally, men may not be encouraged to express vulnerability or seek emotional support (62), thus, when confronted with loneliness and the need for social interaction, they might prefer to opt for low-risk, non-direct interpersonal methods, such as engaging with conversational AI to alleviate feelings of loneliness.

Conversely, females under depressive states were more likely to seek companionship through conversational AI. This behavior could be understood in light of women’s generally higher emotional sensitivity and greater expectations for the quality of social interactions (63). Women are more likely to seek emotional support and understanding when feeling depressed, and AI chatbots offer a safe space free from judgment, where they can express their feelings and receive immediate feedback and comfort.

These gender disparities elucidate the influence of societal norms and expectations on technology utilization. Men might perceive conversational AI as a strategic method to mitigate feelings of loneliness without the necessity to overtly articulate their social needs or seek direct interpersonal assistance, thereby bolstering their perceptions of autonomy and self-efficacy. Conversely, women may employ these technologies as adjunctive resources for emotional articulation and social engagement, thereby augmenting their emotional connectivity and gratification derived from social interactions. This delineation underscores the nuanced manner in which gender roles shape the adoption and application of technological solutions in addressing individual psychological conditions (64).

The role of mind perception of conversational AI varies significantly under different psychological states. When mind perception is high, the relationship between depression and UCAI-C becomes more pronounced. This can be explained by the fact that when users perceive AI as having higher cognitive and emotional abilities, they are more likely to trust and rely on these systems for emotional support and understanding (65). A higher level of mind perception may create a more “human-like” interaction experience for individuals with depression, making them more inclined to use these technologies as a source of emotional relief (66).

In contrast, mind perception buffered the positive relationship between loneliness and UCAI-U. This suggests that, in situations where mind perception is lower, users may not expect AI to provide deep emotional or psychological understanding. Instead, they are more likely to seek basic social interaction or companionship (67). In this context, AI chatbots, while potentially offering surface-level or programmed interactions, serve as a readily available social option that provides continuous communication and interaction, which can be effective in alleviating feelings of loneliness (56).

However, given the correlational nature of the present findings, the directionality between mind perception and UCAI-C warrants careful consideration. Although we interpreted mind perception primarily as a factor influencing the decision to engage with conversational AI for emotional support, alternative interpretations are equally plausible. For instance, it is possible that certain individuals attribute greater mental capacities and emotional intelligence to AI chatbots as a consequence of their unmet social needs, loneliness, or depression, thereby enhancing the perceived social value and attractiveness of these tools. Supporting this alternative interpretation, previous studies indicate that people experiencing loneliness or social deficits tend to anthropomorphize AI companions more readily to fulfill their social interaction needs (68). Similarly, recent research highlights that users may strategically ascribe higher consciousness and human-like qualities to AI companions, thereby enabling deeper emotional interactions that can mitigate loneliness and foster social health benefits (69, 70). Thus, the observed correlation between mind perception and increased companionship-oriented chatbot use might also reflect users’ active attempts to compensate for emotional and social deficits by enhancing their perception of AI’s social presence and empathetic capabilities. Future longitudinal and experimental studies are needed to further clarify these directional and potentially reciprocal relationships.

This difference may be closely tied to individual expectations and the purpose for using AI. Users with high mind perception of AI may seek more complex emotional feedback and psychological support from their interactions with AI, and are thus more likely to turn to AI for help when feeling down (71). Conversely, users with lower mind perception may view AI more as a simple communication tool, primarily used to supplement social interactions in daily life and mitigate loneliness, rather than to engage in deep emotional exploration or resolution.

The findings of this study have profound implications for the development and deployment of AI-driven mental health care, especially given the tendency of individuals with depression and loneliness to seek support from AI chatbots. By designing chatbot interactions to more convincingly mimic human empathy and support, these tools could become essential components of mental health management, especially in contexts where direct human interaction is limited. Moreover, recognizing the distinct needs based on gender and mind perception underscores the importance of personalizing chatbot interactions to match user preferences and emotional needs, enhancing their therapeutic potential. However, it is crucial to remain vigilant about the potential for problematic use of companion AI (39), which could exacerbate negative impacts on individuals already experiencing depression or loneliness. This calls for careful monitoring and the implementation of safeguards to prevent dependency and ensure that AI tools serve as beneficial supplements to traditional mental health care (72).

Despite the potential benefits, it is important to remain cautious regarding the problematic aspects of AI companionship use, particularly when loneliness and high mind perception drive dependence or excessive reliance on conversational AI. Recent studies have warned that individuals with higher social anxiety, loneliness, or a tendency toward rumination may be particularly vulnerable to problematic chatbot use, wherein frequent AI interactions exacerbate rather than alleviate their psychological distress (39). Moreover, consumer–machine relationships driven by intense emotional engagement and anthropomorphic tendencies might pose ethical challenges, including dependency risks and reduced motivation for meaningful human interactions (73). Therefore, future developments and applications of AI chatbots must include mechanisms for monitoring usage patterns and providing safeguards against potential over-reliance or negative psychological outcomes.

## 5 Limitations

This study has several limitations. The reliance on self-reported measures introduces the possibility of response biases, which may affect the accuracy of the findings regarding AI usage and psychological states. Additionally, the cross-sectional nature of the study limits the ability to infer causality between depression, loneliness, and AI chatbot use. Longitudinal studies would be beneficial to establish temporal relationships and better understand the dynamics over time. Moreover, the study’s focus on a college student demographic may limit the generalizability of the findings to other age groups or populations with different socio-economic backgrounds. Future research could expand to include a more diverse population to validate these findings across various demographic profiles.

## 6 Future research directions

Future research could extend the current findings by adopting longitudinal and experimental designs, thereby clarifying the causal directions among depression, loneliness, mind perception, and conversational AI use. Such designs would help establish temporal dynamics and better identify mechanisms underlying these relationships. Additionally, investigating the specific conditions and psychological processes under which interactions with conversational AI transition from beneficial to problematic use would be particularly valuable (39, 73). Personalization of chatbot interactions based on individual differences—including gender, psychological state, and mind perception—represents another important area for future studies, potentially enhancing the effectiveness of AI interventions. Finally, expanding the demographic scope beyond college students to include different age groups, diverse cultural contexts, and varied socio-economic backgrounds would significantly improve the generalizability and ecological validity of research findings in this domain.

## 7 Conclusion

This study highlights how depression and loneliness influence the use of AI chatbots for companionship. We found that depression significantly increases usage, with loneliness acting as a key mediator. Additionally, perceptions of the chatbots’ mental capabilities and gender differences also affect this usage. These insights suggest that AI chatbots could be tailored as supportive tools for those with depression, potentially offering a valuable resource for emotional support and social interaction. This research underscores the need to consider individual differences in the design and deployment of AI technologies for mental health support.

## Data Availability

The original contributions presented in the study are included in the article/supplementary material, further inquiries can be directed to the corresponding author. The survey data and analytical code used in this study are openly available on the figshare platform at the following link: https://figshare.com/projects/Depression_and_the_use_of_conversational_AI_for_companionship_among_college_students_The_mediating_role_of_loneliness_and_the_moderating_effects_of_gender_and_mind_perception/245480.

## References

[1] Vzorin GD Bukinich AM Sedykh AV Vetrova II Sergienko EA. The emotional intelligence of the GPT-4 large language model. Psychol Russia. (2024) 17:85–99. doi: 10.11621/pir.2024.0206, PMID: 39552777 PMC11562005

[2] Sorin V Brin D Barash Y Konen E Charney A Nadkarni G. Large language models and empathy: systematic review. J Med Internet Res. (2024) 26:e52597. doi: 10.2196/52597 PMC11669866 39661968

[3] Ayers JW Poliak A Dredze M Leas EC Zhu Z Kelley JB. Comparing physician and artificial intelligence chatbot responses to patient questions posted to a public social media forum. JAMA Intern Med. (2023) 183:589–96. doi: 10.1001/jamainternmed.2023.1838, PMID: 37115527 PMC10148230

[4] Sharma A Lin IW Miner AS Atkins DC Althoff T. Human–AI collaboration enables more empathic conversations in text-based peer-to-peer mental health support. Nat Mach Intell. (2023) 5:46–57. doi: 10.1038/s42256-022-00593-2

[5] Zhao W. Zhao Y. Lu X. Wang S. Tong Y. Qin B. (2023). Is ChatGPT equipped with emotional dialogue capabilities? (no. arXiv:2304.09582). arXiv. Available online at: http://arxiv.org/abs/2304.09582

[6] Yin Y Jia N Wakslak CJ. AI can help people feel heard, but an AI label diminishes this impact. Proc Natl Acad Sci. (2024) 121:e2319112121. doi: 10.1073/pnas.2319112121, PMID: 38551835 PMC10998586

[7] Boucher EM Harake NR Ward HE Stoeckl SE Vargas J Minkel J. Artificially intelligent chatbots in digital mental health interventions: a review. Expert Rev Med Devices. (2021) 18:37–49. doi: 10.1080/17434440.2021.2013200, PMID: 34872429

[8] Koverola M Kunnari A Sundvall J Laakasuo M. General attitudes towards robots scale (GAToRS): a new instrument for social surveys. Int J Soc Robot. (2022) 14:1559–81. doi: 10.1007/s12369-022-00880-3

[9] Denecke K Abd-Alrazaq A Househ M. Artificial intelligence for Chatbots in mental health: opportunities and challenges. In: Househ M Borycki E Kushniruk A, editors. Multiple perspectives on artificial intelligence in healthcare. Cham, Switzerland: Springer International Publishing (2021). 115–28.

[10] Chaturvedi R Verma S Das R Dwivedi YK. Social companionship with artificial intelligence: recent trends and future avenues. Technol Forecast Soc Chang. (2023) 193:122634. doi: 10.1016/j.techfore.2023.122634

[11] Wiederhold BK. The rise of AI companions and the quest for authentic connection. Cyberpsychol Behav Soc Netw. (2024) 27:524–6. doi: 10.1089/cyber.2024.0309, PMID: 38916124

[12] Ali I Warraich NF Butt K. Acceptance and use of artificial intelligence and AI-based applications in education: a meta-analysis and future direction. Inf Dev. (2024) 40:02666669241257206. doi: 10.1177/02666669241257206, PMID: 40372777

[13] Kelly S Kaye S-A Oviedo-Trespalacios O. What factors contribute to the acceptance of artificial intelligence? A systematic review. Telematics Inform. (2023) 77:101925. doi: 10.1016/j.tele.2022.101925, PMID: 40370555

[14] Latikka R. Bergdahl J. Savolainen I. Celuch M. Oksanen A. (2024). A friend or foe? A six-country study of individual and well-being factors associated with Chatbot friend usage. Available online at: https://papers.ssrn.com/sol3/papers.cfm?abstract_id=4806287 (Accessed December 4, 2024).

[15] Hartanto A Quek FY Tng GY Yong JC. Does social media use increase depressive symptoms? A reverse causation perspective. Front Psych. (2021) 12:641934. doi: 10.3389/fpsyt.2021.641934, PMID: 33833700 PMC8021694

[16] Houghton S Lawrence D Hunter SC Rosenberg M Zadow C Wood L. Reciprocal relationships between trajectories of depressive symptoms and screen media use during adolescence. J Youth Adolesc. (2018) 47:2453–67. doi: 10.1007/s10964-018-0901-y, PMID: 30046970 PMC6208639

[17] Chen S-K Lin SS. A latent growth curve analysis of initial depression level and changing rate as predictors of problematic internet use among college students. Comput Hum Behav. (2016) 54:380–7. doi: 10.1016/j.chb.2015.08.018

[18] Teng Z Pontes HM Nie Q Griffiths MD Guo C. Depression and anxiety symptoms associated with internet gaming disorder before and during the COVID-19 pandemic: a longitudinal study. J Behav Addict. (2021) 10:169–80. doi: 10.1556/2006.2021.00016, PMID: 33704085 PMC8969853

[19] Heffer T Good M Daly O MacDonell E Willoughby T. The longitudinal association between social-media use and depressive symptoms among adolescents and young adults: an empirical reply to Twenge et al. (2018). Clin Psychol Sci. (2019) 7:462–70. doi: 10.1177/2167702618812727, PMID: 40372777

[20] Puukko K Hietajärvi L Maksniemi E Alho K Salmela-Aro K. Social media use and depressive symptoms—a longitudinal study from early to late adolescence. Int J Environ Res Public Health. (2020) 17:5921. doi: 10.3390/ijerph17165921, PMID: 32824057 PMC7459880

[21] Kardefelt-Winther D. A conceptual and methodological critique of internet addiction research: towards a model of compensatory internet use. Comput Hum Behav. (2014) 31:351–4. doi: 10.1016/j.chb.2013.10.059

[22] Ait Baha T El Hajji M Es-Saady Y Fadili H. The power of personalization: a systematic review of personality-adaptive Chatbots. SN Comput Sci. (2023) 4:661. doi: 10.1007/s42979-023-02092-6

[23] Rostami M Navabinejad S. Artificial empathy: user experiences with emotionally intelligent Chatbots. AI Tech Behav Soc Sci. (2023) 1:19–27. doi: 10.61838/kman.aitech.1.3.4

[24] Velagaleti SB Choukaier D Nuthakki R Lamba V Sharma V Rahul S. Empathetic algorithms: the role of AI in understanding and enhancing human emotional intelligence. J Electr Syst. (2024) 20:2051–60. doi: 10.52783/jes.1806

[25] Ge L Yap CW Ong R Heng BH. Social isolation, loneliness and their relationships with depressive symptoms: a population-based study. PLoS One. (2017) 12:e0182145. doi: 10.1371/journal.pone.0182145, PMID: 28832594 PMC5568112

[26] Peplau L. A. Perlman D. (Eds.). (1982). Loneliness: a sourcebook of current theory, research, and therapy. New York: Wiley. Available online at: https://cir.nii.ac.jp/crid/1130282270437919360 (Accessed December 4, 2024)

[27] Erzen E Çikrikci Ö. The effect of loneliness on depression: a meta-analysis. Int J Soc Psychiatry. (2018) 64:427–35. doi: 10.1177/0020764018776349, PMID: 29792097

[28] Achterbergh L Pitman A Birken M Pearce E Sno H Johnson S. The experience of loneliness among young people with depression: a qualitative meta-synthesis of the literature. BMC Psychiatry. (2020) 20:415. doi: 10.1186/s12888-020-02818-3, PMID: 32831064 PMC7444250

[29] Lasgaard M Goossens L Elklit A. Loneliness, depressive symptomatology, and suicide ideation in adolescence: cross-sectional and longitudinal analyses. J Abnorm Child Psychol. (2011) 39:137–50. doi: 10.1007/s10802-010-9442-x, PMID: 20700646

[30] Van As BAL Imbimbo E Franceschi A Menesini E Nocentini A. The longitudinal association between loneliness and depressive symptoms in the elderly: a systematic review. Int Psychogeriatr. (2022) 34:657–69. doi: 10.1017/S1041610221000399, PMID: 33849675

[31] Cacioppo JT Cacioppo S Boomsma DI. Evolutionary mechanisms for loneliness. Cognit Emot. (2014) 28:3–21. doi: 10.1080/02699931.2013.837379, PMID: 24067110 PMC3855545

[32] Qualter P Vanhalst J Harris R Van Roekel E Lodder G Bangee M. Loneliness across the life span. Perspect Psychol Sci. (2015) 10:250–64. doi: 10.1177/1745691615568999, PMID: 25910393

[33] Beacker R. Sellen K. Crosskey S. Boscart V. Barbosa Neves B. (2014). Technology to reduce social isolation and loneliness. Proceedings of the 16th international ACM SIGACCESS conference on computers & accessibility - ASSETS 14, 27–34.

[34] Scott RA Zimmer-Gembeck MJ Gardner AA Hawes T Modecki KL Duffy AL. Daily use of digital technologies to feel better: adolescents’ digital emotion regulation, emotions, loneliness, and recovery, considering prior emotional problems. J Adolesc. (2024) 96:539–50. doi: 10.1002/jad.12259, PMID: 37811912

[35] Margalit M. Loneliness and virtual connections In: Margalit IM, editor. Lonely children and adolescents. New York: Springer (2010). 171–99.

[36] Balcombe L De Leo D. The impact of YouTube on loneliness and mental health. Informatics. (2023) 10:39. doi: 10.3390/informatics10020039

[37] Rodríguez-Martínez A Amezcua-Aguilar T Cortés-Moreno J Jiménez-Delgado JJ. Qualitative analysis of conversational Chatbots to alleviate loneliness in older adults as a strategy for emotional health. Healthcare. (2023) 12:62. doi: 10.3390/healthcare12010062, PMID: 38200967 PMC10779105

[38] Skjuve M Følstad A Fostervold KI Brandtzaeg PB. My chatbot companion-a study of human-chatbot relationships. Int J Hum Comput Stud. (2021) 149:102601. doi: 10.1016/j.ijhcs.2021.102601

[39] Hu B Mao Y Kim KJ. How social anxiety leads to problematic use of conversational AI: the roles of loneliness, rumination, and mind perception. Comput Hum Behav. (2023) 145:107760. doi: 10.1016/j.chb.2023.107760

[40] Strzelecki A ElArabawy S. Investigation of the moderation effect of gender and study level on the acceptance and use of generative AI by higher education students: comparative evidence from Poland and Egypt. Br J Educ Technol. (2024) 55:1209–30. doi: 10.1111/bjet.13425

[41] Mao Y Liu S Ni Q Lin X He L. A review on machine theory of mind. IEEE Trans Comput Soc Syst. (2024) 11:7114–32. doi: 10.1109/TCSS.2024.3416707

[42] Koban K Banks J. It feels, therefore it is: associations between mind perception and mind ascription for social robots. Comput Hum Behav. (2024) 153:108098. doi: 10.1016/j.chb.2023.108098

[43] Astobiza AM. Do people believe that machines have minds and free will? Empirical evidence on mind perception and autonomy in machines. AI Ethics. (2024) 4:1175–83. doi: 10.1007/s43681-023-00317-1

[44] Kocalevent R-D Hinz A Brähler E. Standardization of the depression screener patient health questionnaire (PHQ-9) in the general population. Gen Hosp Psychiatry. (2013) 35:551–5. doi: 10.1016/j.genhosppsych.2013.04.006, PMID: 23664569

[45] Manea L Gilbody S McMillan D. Optimal cut-off score for diagnosing depression with the patient health questionnaire (PHQ-9): a meta-analysis. CMAJ. (2012) 184:E191–6. doi: 10.1503/cmaj.110829, PMID: 22184363 PMC3281183

[46] Xiao R Du J. Reliability and validity of the 6-item UCLA loneliness scale (ULS-6) for application in adults. Nan fang yi ke da xue xue bao. J South Med Univ. (2023) 43:900–5. 10.12122/j.issn.1673-4254.2023.06.04 PMC10339310 37439161

[47] Gray HM Gray K Wegner DM. Dimensions of mind perception. Science. (2007) 315:619–9. doi: 10.1126/science.1134475, PMID: 17272713

[48] Stafford RQ MacDonald BA Jayawardena C Wegner DM Broadbent E. Does the robot have a mind? Mind perception and attitudes towards robots predict use of an eldercare robot. Int J Soc Robot. (2014) 6:17–32. doi: 10.1007/s12369-013-0186-y

[49] Bao H.-W.-S. (2022). bruceR: broadly useful convenient and efficient R functions. R package version 0.8. X. Available online at: https://cran.r-project.org/package=bruceR (Accessed November 10, 2024).

[50] Muller D Judd CM Yzerbyt VY. When moderation is mediated and mediation is moderated. J Pers Soc Psychol. (2005) 89:852–63. doi: 10.1037/0022-3514.89.6.852, PMID: 16393020

[51] Frison E Eggermont S. The impact of daily stress on adolescents’ depressed mood: the role of social support seeking through Facebook. Comput Hum Behav. (2015) 44:315–25. doi: 10.1016/j.chb.2014.11.070

[52] Epkins CC Heckler DR. Integrating etiological models of social anxiety and depression in youth: evidence for a cumulative interpersonal risk model. Clin Child Fam Psychol Rev. (2011) 14:329–76. doi: 10.1007/s10567-011-0101-8, PMID: 22080334

[53] Hames JL Hagan CR Joiner TE. Interpersonal processes in depression. Annu Rev Clin Psychol. (2013) 9:355–77. doi: 10.1146/annurev-clinpsy-050212-185553, PMID: 23297787

[54] Gao Y Li A Zhu T Liu X Liu X. How smartphone usage correlates with social anxiety and loneliness. PeerJ. (2016) 4:e2197. doi: 10.7717/peerj.2197, PMID: 27478700 PMC4950540

[55] Hawkley LC Cacioppo JT. Loneliness matters: a theoretical and empirical review of consequences and mechanisms. Ann Behav Med. (2010) 40:218–27. doi: 10.1007/s12160-010-9210-8, PMID: 20652462 PMC3874845

[56] De Freitas J Uğuralp AK Uğuralp Z Puntoni S. AI companions are perceived to be backed by humans and this attenuates loneliness. *Nat Human Behav*. (2024) 8:646–56. doi: 10.1038/s41562-023-01795-1

[57] Pani B Crawford J Allen K-A. Can generative artificial intelligence Foster belongingness, social support, and reduce loneliness? A conceptual analysis In: Lyu Z, editor. Applications of generative AI. Cham: Springer International Publishing (2024). 261–76.

[58] Maples B Cerit M Vishwanath A Pea R. Loneliness and suicide mitigation for students using GPT3-enabled chatbots. NPJ Ment Health Res. (2024) 3:4. doi: 10.1038/s44184-023-00047-6, PMID: 38609517 PMC10955814

[59] Alotaibi JO Alshahre AS. The role of conversational AI agents in providing support and social care for isolated individuals. Alex Eng J. (2024) 108:273–84. doi: 10.1016/j.aej.2024.07.098

[60] Chaplin TM. Gender and emotion expression: a developmental contextual perspective. Emot Rev. (2015) 7:14–21. doi: 10.1177/1754073914544408, PMID: 26089983 PMC4469291

[61] Chaplin TM Aldao A. Gender differences in emotion expression in children: a meta-analytic review. Psychol Bull. (2013) 139:735–65. doi: 10.1037/a0030737, PMID: 23231534 PMC3597769

[62] Keohane A Richardson N. Negotiating gender norms to support men in psychological distress. Am J Mens Health. (2018) 12:160–71. doi: 10.1177/1557988317733093, PMID: 29019282 PMC5734549

[63] Fischer AH Kret ME Broekens J. Gender differences in emotion perception and self-reported emotional intelligence: a test of the emotion sensitivity hypothesis. PLoS One. (2018) 13:e0190712. doi: 10.1371/journal.pone.0190712, PMID: 29370198 PMC5784910

[64] Kim A Cho M Ahn J Sung Y. Effects of gender and relationship type on the response to artificial intelligence. Cyberpsychol Behav Soc Netw. (2019) 22:249–53. doi: 10.1089/cyber.2018.0581, PMID: 30864826

[65] Lee I Hahn S. On the relationship between mind perception and social support of chatbots. Front Psychol. (2024) 15:1282036. doi: 10.3389/fpsyg.2024.1282036, PMID: 38510306 PMC10952123

[66] Dosovitsky G Pineda BS Jacobson NC Chang C Bunge EL. Artificial intelligence chatbot for depression: descriptive study of usage. JMIR Form Res. (2020) 4:e17065. doi: 10.2196/17065, PMID: 33185563 PMC7695525

[67] Merrill K Kim J Collins C. AI companions for lonely individuals and the role of social presence. Commun Res Rep. (2022) 39:93–103. doi: 10.1080/08824096.2022.2045929

[68] Eyssel F. Reich N. (2013). Loneliness makes the heart grow fonder (of robots): on the effects of loneliness on psychological anthropomorphism. Proceedings of the 8th ACM/IEEE international conference on human-robot interaction, 121–122.

[69] Guingrich RE Graziano MSA. Ascribing consciousness to artificial intelligence: human-AI interaction and its carry-over effects on human-human interaction. Front Psychol. (2024) 15:1322781. doi: 10.3389/fpsyg.2024.1322781, PMID: 38605842 PMC11008604

[70] Guingrich RE Graziano MSA. Chatbots as social companions: how people perceive consciousness, human likeness, and social health benefits in machines. Oxford, UK: xford University Press (2024).

[71] Shank DB Graves C Gott A Gamez P Rodriguez S. Feeling our way to machine minds: People’s emotions when perceiving mind in artificial intelligence. Comput Hum Behav. (2019) 98:256–66. doi: 10.1016/j.chb.2019.04.001

[72] Cabrera J Loyola MS Magaña I Rojas R. Ethical dilemmas, mental health, artificial intelligence, and LLM-based Chatbots In: Rojas I Valenzuela O Rojas Ruiz F Herrera LJ Ortuño F, editors. Bioinformatics and biomedical engineering, vol. 13920. Cham, Switzerland: Springer Nature Switzerland (2023). 313–26.

[73] Pentina I Xie T Hancock T Bailey A. Consumer–machine relationships in the age of artificial intelligence: systematic literature review and research directions. Psychol Mark. (2023) 40:1593–614. doi: 10.1002/mar.21853

